# Plasma pentraxin 3 is higher in early ovarian hyperstimulation syndrome than in uncomplicated in vitro fertilization cycle of high-risk women

**DOI:** 10.1007/s00404-020-05556-9

**Published:** 2020-05-05

**Authors:** Kati Korhonen, Leila Unkila-Kallio, Henrik Alfthan, Esa Hämäläinen, Aila Tiitinen, Tomi Mikkola, Juha Tapanainen, Hanna Savolainen-Peltonen

**Affiliations:** 1 Obstetrics and Gynecology, University of Helsinki and Helsinki University Hospital, Haartmaninkatu 2, PO Box 140, 00290 Helsinki, Finland; 2 HUSLAB, Helsinki University Hospital, Topeliuksenkatu 32, 00290 Helsinki, Finland; 3 Clinical Chemistry, University of Helsinki and Helsinki University Hospital, Haartmaninkatu 8, 00290 Helsinki, Finland; 4 Folkhälsan Research Center, Biomedicum Helsinki, Haartmaninkatu 8, 00290 Helsinki, Finland; 5 PEDEGO Research Unit, Medical Research Center, Obstetrics and Gynecology, University of Oulu and Oulu University Hospital, Pentti Kaiteran katu 1, Linnanmaa, 90014 Oulu, Finland

## Abstract

**Purpose:**

Pentraxin 3 (PTX3) is a locally secreted, quicker responsive pro-inflammatory protein than C-reactive protein (CRP). We evaluated the value of PTX3 in the prediction of ovarian hyperstimulation syndrome (OHSS), a severe complication of in vitro fertilization (IVF).

**Methods:**

This two-year prospective follow-up study included 27 women with uncomplicated IVF-cycles (IVF group) and 31 patients diagnosed with moderate or severe early OHSS (OHSS group). PTX3 was analysed from follicular fluid (FF) and serial blood samples with enzyme-linked immunoassay and CRP with particle-enhanced immunoturbidimetric assay. The value of PTX3 and CRP in detecting OHSS was examined with receiver operating characteristic (ROC) curve analysis and expressed as the area under the curve (AUC).

**Results:**

The circulating PTX3 level peaked at two days after oocyte pick-up (OPU2), and in the OHSS group the level was 1.9 times higher (*P* = 0.006) than in the IVF group. However, in ROC curve analysis PTX3 (AUC 0.79, best cut off 1.1 µg/L) was not superior to CRP (AUC 0.87; best cut off 9.5 mg/L) in predicting early OHSS. In the IVF group, the FF-PTX3 concentration was 15–20 times higher than in the plasma. PTX3 level at OPU2 correlated with the number of punctured follicles (*r* = 0.56, *n* = 22, *P* = 0.006). Triggering with human chorionic gonadotrophin or early pregnancy had no effect on PTX3 level.

**Conclusion:**

The elevated PTX3 concentration in OHSS at OPU2, when freeze-all embryos strategy is still possible to consider, indicates that PTX3 level could provide additional benefit in the risk assessment for early OHSS.

## Introduction

Ovarian hyperstimulation syndrome (OHSS) is the most severe complication of in vitro fertilization (IVF) [1, 2] and inflammation, endothelial dysfunction and increased capillary permeability play a profound role in its pathophysiology. The concentrations of several proinflammatory cytokines and C-reactive protein (CRP) are increased in OHSS [3–5], but none of them has so far been shown useful in predicting the syndrome. Instead, anti-Müllerian hormone (AMH) and the antral follicle count are the most commonly used tools to predict the risk for OHSS [6–9].

Pentraxin 3 (PTX3) is a long chain recognition molecule participating in human innate immunity system [10]. Unlike the liver-produced CRP, PTX3 is secreted i.e. from activated endothelial cells and macrophages, but also from the ovarian granulosa cells [11–13]. High PTX3 concentration has been associated with endothelial dysfunction [14], and increasing data indicate that PTX3 is a prognostic factor for diverse clinical conditions, such as acute and chronic heart diseases [15, 16], sepsis [17–19] and pre-eclampsia [20]. Due to the local production in the injured tissue, circulating PTX3 levels increase more rapidly than CRP during an inflammatory process [10, 21].

Both PTX3 and AMH are secreted from the ovarian granulosa cells: AMH from small antral follicles [22] and PTX3 from antral and periovulatory follicles [12, 23]. Moreover, the same oocyte-derived factors, namely growth differentiation factor-9 (GDF9) and bone morphogenic proteins (BMP) [24–26] regulate the synthesis of PTX3 and AMH. During the natural menstrual cycle, circulating PTX3 levels are higher during the early follicular (menstruation) than the luteal phase [27]. In addition, human chorionic gonadotrophin (hCG) and follicle-stimulating hormone (FSH) induce PTX3 expression in the human cumulus oophorus matrix and granulosa cells [12, 23]. Thus, tissue-specific PTX3 secretion from the follicles during IVF stimulation could be an early marker of inflammation and high ovarian response and thus reflect the risk for OHSS. Thus far, the knowledge about PTX3 during the IVF cycle is scarce. One small study has shown unchanged PTX3 concentration between the start of an antagonist IVF cycle and oocyte pick-up (OPU) [28]. In addition, systemic PTX3 level at the time of blastocyst transfer has been indicated as a possible biomarker for IVF success [29].

PTX3 crosslinks immunity, inflammation and reproduction [30], and together with high AMH level, may be linked to the multifollicular response in IVF cycle and especially in OHSS. Our primary aim was to study whether circulating PTX3 level could be used for the prediction of early OHSS. Furthermore, the timing of PTX3 response during an uncomplicated IVF cycle, OHSS and during the recovery from OHSS was compared with the traditionally used inflammation marker CRP. Finally, as both PTX3 and AMH originate from granulosa cells, we evaluated whether follicular fluid or plasma AMH concentrations were associated with the corresponding PTX3 levels.

## Materials and methods

The study was a prospective follow-up of IVF patients treated in 2006–2008 at Helsinki University Hospital, Department of Obstetrics and Gynecology, Finland. The whole study protocol has been described previously [3]. For the present study, we included two groups: women undergoing uncomplicated IVF/ICSI cycles and patients with early OHSS. Data on patient characteristics, medical history, infertility treatments, and symptoms were retrieved through a structured questionnaire and medical records. The main outcome measure was the plasma PTX3 level between these study groups.

### Patients and samples

In brief, for the IVF cycle group we recruited 30 generally healthy women carrying clinically estimated, known risk factors for OHSS (previous OHSS, anovulation, polycystic ovary syndrome, Body Mass Index < 20 kg/m^2^, age < 25). Patients with severe endometriosis and only one ovary were excluded. Three of the women developed OHSS and were therefore recruited and transferred to the OHSS group. Thus, the IVF group finally comprised of 27 women who were treated with a long gonadotrophin-releasing hormone (GnRH) agonist protocol with hCG triggering, vaginal luteal support and a cleavage-stage embryo transfer [3]. Repeated clinical examinations were done, and serial blood samples were taken as follows: prior to treatment (mid-luteal phase, baseline, *n* = 22), on stimulation day one (suppression, *n* = 21), the day of OPU (*n* = 26), and two to three (OPU2, *n* = 27), six to eight (OPU7, *n* = 19), 13 to 15 days (OPU14, *n* = 22), and five weeks (OPU35, *n* = 18) after OPU. Clear follicle fluid (FF) from the dominant follicles on both sides was collected at OPU (*n* = 23).

For the OHSS group, we recruited patients attending the emergency department due to symptoms of early OHSS (abdominal distension, floating or pain, nausea, dyspnoea, diminished diuresis, fever, ascites or pleural fluid in ultrasound or X-ray imagining), including the three patients originally in the IVF-group (*n* = 41). Patients with suspected ovarian torsion and intra-abdominal haemorrhage were excluded. The physician on duty did a gynaecological and ultrasound examination, an X-ray in the case of dyspnoea, and made the decisions about hospitalizing or discharging the patient, treatment with intravenous fluids and drainage of ascites and pleural fluid. The severity of the syndrome was classified according to internationally accepted criteria [31]. OHSS cases with concomitant infections (fever ≥ 37.5 °C with intense abdominal pain, *n* = 5), and mild OHSS (*n* = 5) were excluded from the study. The women were generally healthy, in other words, they did not have any potentially confounding inflammatory diseases. Finally, the OHSS group comprised of 24 moderate and seven severe early OHSS patients. Serial blood samples were taken: on admission (*n* = 31), repeatedly on the ward, at discharge (*n* = 30), and one week after discharge (surveillance visit, *n* = 24). When paracentesis was indicated, an ascites sample was taken, if possible (*n* = 4). To determine the recovery, we also specified the worst OHSS day under hospitalization based on clinical data (dyspnoea, diminished diuresis, fever, maximum waist circumference, weight, and the maximum drainage of ascites or pleural fluid).

All study samples were centrifugated at 1000 g for 10 min prior storing at -80 °C until analysis. The samples that were taken after OPU were timed as days after OPU (OPUd).

### PTX3, AMH and CRP assays

PTX3 was analysed by enzyme-linked immunoassay (Quantikine ELISA Human Pentraxin 3®/TSG-14 Immunoassay®, R&D Systems, Minneapolis, MN, USA) with the detection limit 0.025 µg/L and the intra- and inter-assay variations (CV%) 3.8% and 6.2%, respectively.

CRP was measured by particle-enhanced immunoturbidimetric assay (Tina-quant C-Reactive Protein Gen.3®, Roche Diagnostics, Rotkreuz, Switzerland) on a Hitachi/Roche Modular Analyzer® (Hitachi Ltd., Tokyo, Japan) in HUSLAB with accredited methods; the detection limit of CRP was 3.0 mg/L.

AMH was analysed by an ultrasensitive AMH ELISA (AnshLabs®, Webster, TX, USA) in collaboration with the Helsinki University Hospital Laboratory (HUSLAB). The detection limit was 0.023 µg/L and the intra- and inter-assay variations (CV%) were ≤ 4.0% and < 4.8%, respectively.

### Statistics

The primary outcome was the difference of plasma PTX3 level between the IVF cycle group and the OHSS group. The power analysis was based on a previous finding that in young, healthy, normal-weight Finnish women the change in PTX3 concentrations during contraceptive use was 0.2 µg/L (SD 0.2 µg/L) [32]. The power analysis revealed that the sample size of ten is sufficient to detect one standard deviation difference in PTX3 level between the IVF and OHSS groups (power 80%, error-α level 0.05).

Data were analysed with SPSS for Windows® (version 23). Shapiro–Wilk test was used for the distribution of the variables. Data are expressed as median with inter-quartile range (IQR) for nonparametric or mean and standard deviation (SD) for parametric distributions, when not otherwise stated. Group differences were analysed by Student’s *t* test for parametric, Mann–Whitney *U* test for nonparametric and Chi-Square test for categorical variables. The Wilcoxon’s signed-rank test with Bonferroni correction was used for repeated measurements when Friedman’s two-way analysis of variance by ranks was significant. Spearmanˈs coefficient test was used for correlations. The value of PTX3 in detecting early OHSS was analysed by receiver operating characteristic (ROC) curve analysis and expressed as the area under the curve (AUC). The level of significance was *P* < 0.05.

### Ethical approval

The study was conducted according to the Declaration of Helsinki and approved by the Ethics committee of Helsinki University Hospital (Dnro 504/E9/05). Patients gave a written informed consent at recruitment.

## Results

### Baseline characteristics

Patients in the OHSS group had higher follicle and oocyte count at OPU and underwent fresh embryo transfer less frequently than women in the IVF group (Table 1). A total of ten (32%) of the OHSS patients had been treated with the antagonist protocol (with hCG triggering) whereas all women in the IVF group with the long agonist protocol. Otherwise, the groups were comparable in their primary patient and treatment characteristics. The baseline PTX3, CRP or AMH levels did not correlate with age or body mass index.

**Table 1:** The patient and treatment characteristics

*n*	IVF	OHSS	*P* value
	27	31	
Age (years)^a^	33.9 (32.1–35.7)	32.6 (30.1–35.1)	0.05
BMI (kg/m^2^)^a^	22.7 (19.2–26.2)	21.6 (19.3–23.9)	0.22
Smoking^b^	2 (7)	1 (3)	0.57
Duration of infertility (years)^a^	4.0 (2.8–5.3)	4.0 (2.3–5.8)	0.83
Ethiology^b^			0.79
Male	4 (15)	6 (19)	
Female	6 (22)	8 (26)	
Combined	7 (26)	8 (26)	
Unexplained	10 (37)	8 (26)	
Anovulation^b^	11 (41)	14 (45)	0.80
Agonist protocol^b^	27 (100)	21 (68)	0.001
Cumulative FSH dose (IU)^a^	1500 (1280–1730)	1300 (1040–1560)	0.09
Follicles at OPU^a^	19 (13–26)	35 (26–46)	< 0.001
Oocyte count at OPU^a^	11 (8–15)	20 (14–27)	< 0.001
Embryo transfer^b^	26 (96)	18 (58)	0.001
hCG + /ET^b^	12 (46)	7 (39)	0.76
Baseline AMH (µg/L)	7.0 (2.7–11.2)^a^	10.9–18.4^c^	–

Group differences were analysed by using Mann–Whitney U tests; Chi-Square test for categorical variables

*IVF* in vitro fertilization; *OHSS* ovarian hyperstimulation syndrome; *BMI* body-mass index; *FSH* follicle stimulating hormone; *IU* international units; *OPU* oocyte pick-up; *hCG* + positive pregnancy test at OPU + 14 days; *ET* embryo transfer; *AMH* anti-Müllerian hormone

^a^The data are expressed as median (inter-quartile range)

^b^The data are expressed as the number of patients (percentages)

^c^The data are expressed as range, *n* = 3

### Plasma PTX3, CRP and AMH during uncomplicated IVF cycle

**Fig. 1:**
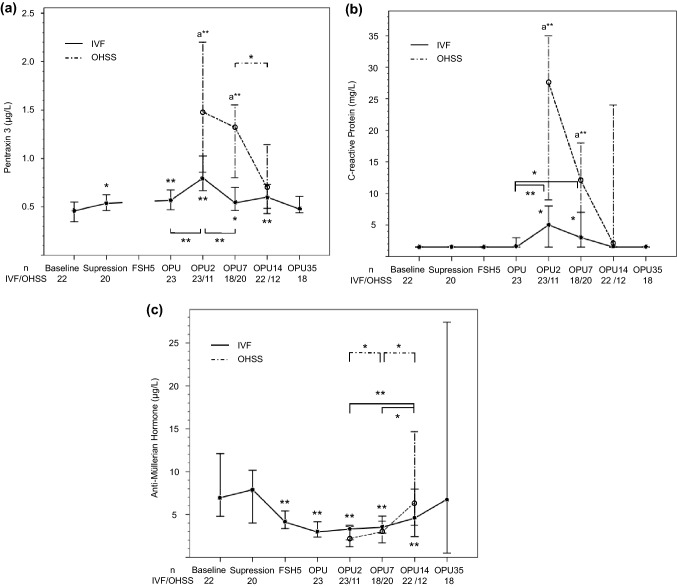
**a** Plasma pentraxin 3 **b** C-reactive Protein and **c** anti-Müllerian hormone concentrations during uncomplicated in vitro fertilization (IVF) cycle and early ovarian hyperstimulation syndrome (OHSS). The data are expressed as medians and 95% confidence intervals. Pairwise comparisons were done with Wilcoxon Signed Rank test (Friedman’s presumption was significant) with Bonferroni correction: *P < 0.05, **P ≤ 0.01 (compared with baseline, when not otherwise stated); and a**P ≤ 0.01, Mann–Whitney *U* test between IVF cycle and OHSS. OPUd, days after the oocyte pick-up

PTX3 concentration increased from baseline (0.46; 0.35–0.56 µg/L) to OPU and peaked at OPU2 (0.80; 0.59–1.0 µg/L, *P* < 0.001 compared to baseline) (Fig. 1a). At the mid-luteal phase of the IVF cycle (OPU7), the level (0.54; 0.42–0.67 µg/L) was higher than at the mid-luteal phase of natural cycle i.e. baseline (*P* < 0.05). PTX3 returned to the baseline level by OPU35. The PTX3 level at OPU2 correlated with the follicle count (*r* = 0.56, *n* = 22, *P* = 0.006), but the baseline AMH level correlated with PTX3 only at OPU7 (*r* = 0.66, *n* = 15, *P* = 0.007).

The CRP level increased from baseline (< 3 mg/L) to OPU2 (5.0; < 3–8.9 mg/L, *P* < 0.01) and returned to the baseline level at OPU14 (Fig. 1b). No correlation between the CRP level and follicle count existed, but CRP correlated with the PTX3 level at OPU2 (*r* = 0.51, *n* = 23, *P* = 0.01) and at OPU7 (*r* = 0.63, *n* = 18, *P* = 0.005).

The AMH level (Table 1, Fig. 1c) was unchanged from baseline to suppression but declined during the stimulation until OPU (3.0; 1.5–4.4 µg/L, *P* < 0.01), remained low up to OPU14 and returned to the baseline level again at OPU35.

The PTX3 level was not different between women with a positive (0.65; 0.45–0.86 µg/L, *n* = 11) and negative (0.54; 0.44–0.64 µg/L, *n* = 11) hCG test at OPU14 (*P* = 0.52) or between women with and without an ongoing pregnancy at OPU35 (*P* = 0.10).

### Plasma PTX 3, CRP and AMH in early OHSS

**Fig. 2:**
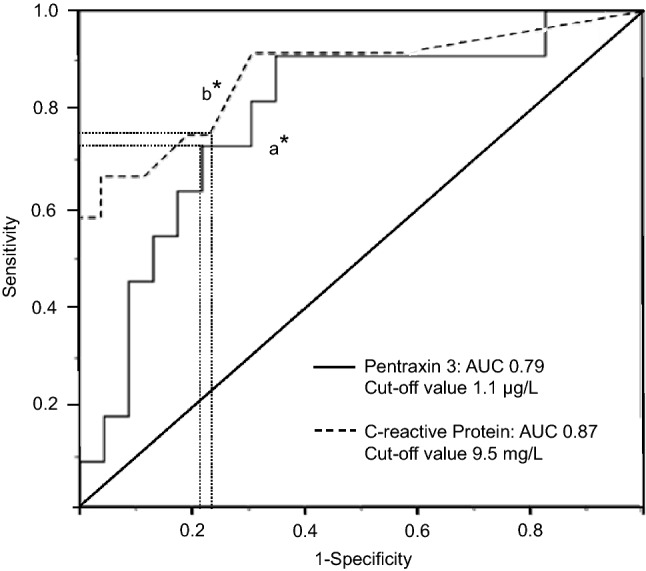
Receiver operating curves of plasma pentraxin 3 and C-reactive protein for detecting early ovarian hyperstimulation syndrome (OHSS), two to three days after oocyte pick-up. Sensitivity and specificity for detecting early OHSS were a* 72% and 79% for PTX3 and b* 75% and 77% for CRP respectively. AUC, area under the curve

In the OHSS group, the PTX3 level was 1.9 times higher at OPU2 (*P* = 0.006) and 2.4 times higher at OPU7 (*P* = 0.001) than in the IVF group (Fig. 1a). The CRP level was also higher in the OHSS than in the IVF group both at OPU2 and at OPU7 (Fig. 1b), but no correlation existed with the PTX3 level (*r* = –0.09, *P* = 0.87, *n* = 6, OPU2). The PTX3 level was not different in patients treated with agonist or antagonist protocol (1.02; 0.59–1.45 µg/L, *n* = 13 vs. 1.39; 0.97–2.24 µg/L, *n* = 7, respectively, *P* = 0.28, OPU7). According to the ROC analysis, the plasma PTX3 level at OPU2 was not better than CRP for predicting early OHSS: the AUCs of the best cut-offs for PTX3 were 0.79 (*P* = 0.007) and for CRP 0.87 (*P* < 0.001) (Fig. 2). The AMH concentrations after OPU did not differ between the IVF and OHSS groups (Fig. 1c).

**Fig. 3:**
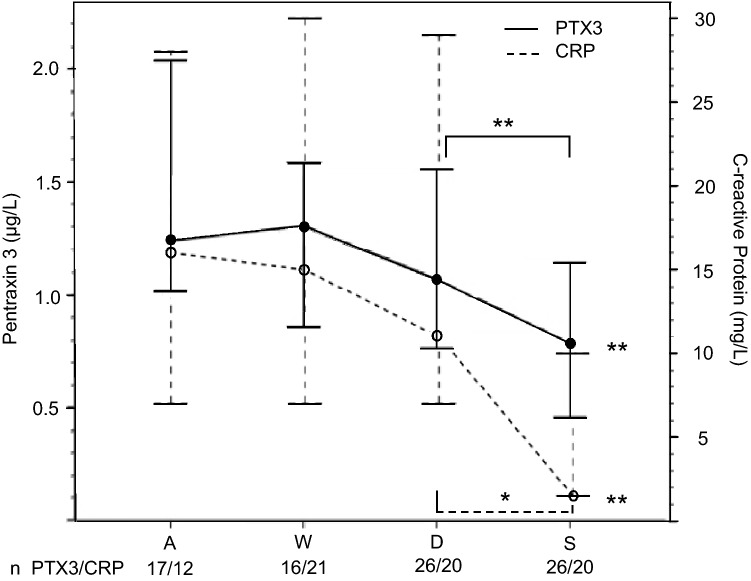
Plasma pentraxin 3 (PTX3) and C-reactive protein (CRP) levels during the recovery from early ovarian hyperstimulation syndrome (OHSS). The time points on *X* axis: *A* on admission to the emergency department; *W* the day of worst symptoms; *D* at discharge from the hospital; *S* at the surveillance visit one week after the discharge. The data are expressed as medians and 95% confidence intervals. Pairwise comparisons were done with Wilcoxon Signed Rank test (Friedman’s presumption was significant): *P < 0.05; **P ≤ 0.01, compared with the value on admission, when not otherwise stated

On admission to the emergency department (OPU1-8), the levels of PTX3 were higher in the OHSS group (1.24; 0.73–1.75 µg/L) than in the IVF group at a similar time (0.69; 0.50–0.88 µg/L, *P* < 0.001). The PTX3 level was the highest in severe OHSS (1.52; 1.06–1.99 µg/L, *n* = 7, OPU7). During treatment on ward, PTX3 level decreased along with the recovery of the symptoms and was the lowest at the follow-up visit. The PTX3 profile during recovery from OHSS was similar to that of CRP (Fig. 3).

### PTX3 and AMH in the follicular fluid and PTX3 in ascites

**Fig. 4:**
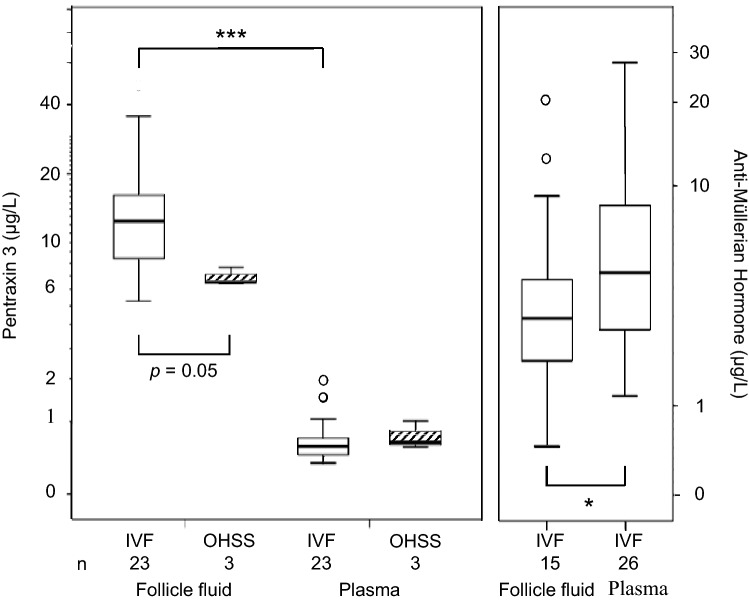
Follicle fluid and plasma pentraxin 3 and anti-Müllerian hormone levels at oocyte pick-up in uncomplicated IVF cycle and early ovarian hyperstimulation syndrome (OHSS). The data are expressed as medians (line inside the box), inter-quartile range (the box), the first and fourth quartile (whiskers). Logarithmic scale on *Y*-axis. Open dots represent outliers. Comparisons between groups are done with Mann–Whitney *U* test or Wilcoxon signed Rank test: *P < 0.05; **P ≤ 0.001

In the IVF group, FF-PTX3 level (12.41; 8.14–16.68 µg/L) was 15–25 times higher than in plasma at OPU (Fig. 4). FF-PTX3 did not correlate with plasma PTX3 (*r* = 0.28, *n* = 13, *P* = 0.4) or with the follicle count (*r* = 0.13, *n* = 22, *P* = 0.6). In the OHSS group, FF was available from the three patients excluded from the IVF group when developing OHSS. Their FF-PTX3 levels tended to be lower than the level in the IVF group (median 6.55; range 6.45–7.65 µg/L, *P* = 0.05), although their plasma PTX3 levels up to OPU were similar to the final IVF group, and after OPU similar to the other OHSS patients (data not shown). In ascites, PTX3 concentrations (median 10.26 µg/L; range 7.14–38.51 µg/L, *n* = 4 samples, OPU6-7) were similar to FF-PTX3.

In the IVF group, FF-AMH level (4.6, < 0.02–9.6 µg/L) was lower than the plasma level at OPU (*P* = 0.02) (Fig. 4), and the FF and plasma levels correlated positively (*r* = 0.60, *n* = 15, *P* = 0.01). No correlation was found between FF-AMH and FF-PTX3 (*r* = 0.15, *n* = 14, *P* = 0.62).

## Discussion

To our knowledge, this is the first study to evaluate the value of circulating PTX3 in the prediction of early OHSS. We showed that the plasma PTX3 levels were higher in early OHSS than during the uncomplicated IVF cycle, which supports the inflammatory nature of early OHSS. Although PTX3 is known as a rapid marker for inflammation, the ROC analysis showed that PTX3 was not better than CRP in predicting early OHSS at OPU2. Moreover, the response to ovarian stimulation and recovery from OHSS was very similar to that of CRP. A novel, but very preliminary finding was the decreased follicular fluid PTX3 level in women about to develop early OHSS. This finding needs to be verified in larger study populations.

### The effect of pituitary suppression, gonadotrophins and OHSS on PTX3

We detected an increase in plasma PTX3 concentration at suppression after GnRH-agonist treatment, but during gonadotrophin stimulation the level was stable. An increase in PTX3 level has been found also during ovarian suppression with contraceptive pills [32]. Both contraceptive pills and long GnRH-agonist treatment reduce the levels of circulating androgens. On the other hand, hyperandrogenism has been associated with low PTX3 levels [33]. Thus, an elevation of the PTX3 level at suppression in the IVF group could be expected. The stable PTX3 level during FSH stimulation is in accordance to the previous study evaluating the PTX3 level between the start of FSH stimulation and OPU, but in that study an antagonist protocol was used [28].

The peak in the PTX3 level at OPU2 may be related, not only to the inflammatory response and tissue damage caused by OPU but also to repair and healing of the ovary as well as the early formation of corpora lutei [34, 35]. The greater PTX3 response in early OHSS than in the uncomplicated IVF cycle probably reflects the larger number of punctured follicles with an increased secretion of PTX3 from e.g. fibroblasts and the endothelial cells. This was supported by the positive correlation between the follicle count and PTX3 level at OPU2. On the other hand, secretion of PTX3 from granulosa cells seems to play a minor role during controlled ovarian hyperstimulation, since FF-PTX3 did not correlate with the circulating levels.

Previous studies [12, 23] indicate that hCG triggering induces the circulating levels of PTX3 at OPU. Whether circulating hCG plays a role in PTX3 regulation during normal pregnancy is unclear. Some studies indicate that the PTX3 levels are elevated during the first trimester of pregnancy [36], whereas in other studies, the level increases with advancing gestational age, and the peak occurs before delivery [37, 38]. In this prospective follow-up, we found no significant effect with hCG triggering or early trophoblastic hCG secretion on PTX3 concentrations up to the seventh week of pregnancy. Thus, it is plausible that PTX3 expression is upregulated in the decidualized endometrium by other factors, such as inflammatory mediators produced by the invading trophoblasts, rather than by hCG [39, 40].

### PTX3 in follicular fluid

The higher FF-PTX3 than plasma PTX3 level in the IVF cycle was in accordance with previous studies [11, 28], and possibly reflects the periovulatory expression and production of PTX3 [12, 23]. Our novel pilot finding was the low FF-PTX3 level in women about to develop early OHSS, in spite of their similar plasma PTX3 levels to the IVF group before OPU. We admit the very small sample size but may speculate that the low FF-PTX3 probably results from the leakage of PTX3 from follicles due to the increased endothelial permeability in women about to develop OHSS. Another possibility is a decreased secretion of PTX3 from the follicular endothelial or granulosa cells into the follicles. PTX3 is known to have a protective role in severe infections and it is involved in tissue repair [34, 35]. Thus, a low FF-PTX3 level could indicate a diminished local ovarian defence against the inflammation and impaired repair capacity after oocyte retrieval, and thus a higher risk for early OHSS.

### PTX3 in relation to AMH

Circulating PTX3 and AMH levels showed notably different profiles during the IVF cycle, although both are secreted by granulosa cells and regulated by the same oocyte derived factors [25]. Several explanations for this finding may exist. Contrary to PTX3, granulosa cells are the only known origin of circulating AMH, and this was supported by the correlation between FF-AMH and plasma level at OPU. Moreover, AMH levels did not differ between early OHSS and IVF cycle after OPU, which suggested that AMH secretion was not affected by the strong inflammatory process seen in early OHSS. The timing may also play a role: AMH is secreted from small antral follicles whereas PTX3 is the major component of the cumulus oophorus complex [22, 23]. As PTX3 correlated positively with the baseline AMH level quite late, one week after OPU, this could reflect the role of PTX3 in the long-lasting healing process of the ovary after OPU.

### The physiological level of PTX3 in infertile women

The commonly used physiological upper limit (2.0 µg/L) for circulating PTX3 is based on cardiovascular studies including both female and male elderly study subjects [10, 41]. However, in later studies with healthy, normal-weight, fertile Caucasian women, PTX3 concentrations have been 1.0 µg/L or less [29, 32, 33]. PTX3 levels may increase by age [18, 42] and obesity [43]. Women in our study were all lean, relatively young and healthy, and their baseline PTX3 levels in the luteal phase of the menstrual cycle were less than 0.5 µg/L. Even during the IVF cycle with multiple ovulations and a stronger inflammatory response than in natural cycle [3, 4], the plasma PTX3 level rarely increased above 1.0 µg/L, and in OHSS rarely above 2.0 µg/L. Thus, our findings, indicate that the upper reference limit for P-PTX3 level needs to be re-evaluated in fertile-age women.

In ROC curve analysis, the best cut off for predicting early OHSS was 1.1 µg/L. This was also near the median circulating level in OHSS patients on admission to the hospital. Before being able to propose the use of PTX3 in the clinical decision-making for the freeze-all embryos strategy, a prospective follow up study with a focus on the timing between OPU and embryo transfer is needed.

### Strengths and limitations

The strength of our study is the detailed prospective follow-up of the IVF cycle and of early OHSS patients until recovery. The frequent sampling gave us the opportunity to thoroughly explore the behaviour of PTX3 and also the effect of gonadotrophins and early pregnancy on PTX3 production. Women in the IVF group were treated with an individualised dose long agonist protocol according to the prevailing practise in Finland during the study period. The agonist protocol is expected to induce a higher inflammatory response than the antagonist protocol [44], and thus, we could expect to find the highest possible circulatory PTX3 response in these cycles to be compared with early OHSS, especially as in both protocols a hCG trigger for ovulation was still used at that time. Moreover, to be able to assess the effect of inflammation on PTX3, patients with concomitant infections as well as other complications than early OHSS were excluded. Further, we used the latest OHSS criteria for categorizing the OHSS group [45].

As a limitation, we used only GnRH-agonist cycles in ovarian stimulation in the IVF group. However, the stimulation protocol did not have any effect on plasma PTX3 levels in OHSS. Second, we could have missed a minor difference between PTX3 and CRP responses, if there is any, as we did not have blood samples between OPU and OPU2. In other clinical conditions such as myocardial infarction, PTX3 peaked in less than 12 h, whereas the CRP level peaked after 24 h after admission to the hospital [41]. We also admit the low number of patients in some subgroup analysis. According to the power analysis, a total of ten patients was a sufficient number for the primary outcome, but this might not apply for the secondary outcomes or subgroup analyses. Finally, as all our participants were Caucasian, the results may not apply to other ethnicities.

## Conclusion

Although plasma PTX3 was higher in early OHSS than in the uncomplicated IVF cycle, it was not superior to the traditionally used inflammatory marker CRP in predicting early OHSS. However, the PTX3 level in the OHSS group peaked at OPU2, which would still give time to consider freezing all embryos to minimize the risk of severe OHSS. Thus, the plasma PTX3 level two days after OPU may be useful in the risk assessment for early OHSS. The pilot finding of low FF-PTX3 at OPU in women about to develop OHSS could indicate its use as a possible early marker of incipient early OHSS if the finding is verified in larger series.
